# 4-(1*H*-Tetra­zol-5-yl)pyridinium bromide

**DOI:** 10.1107/S1600536810050658

**Published:** 2010-12-08

**Authors:** Wen-Ni Zheng, Xin-Yuan Chen

**Affiliations:** a Ordered Matter Science Research Center, College of Chemistry and Chemical Engineering, Southeast University, Nanjing 210096, People’s Republic of China

## Abstract

In the cation of the title compound, C_6_H_6_N_5_^+^·Br^−^, the pyridine and tetra­zole rings are nearly coplanar, forming a dihedral angle of 6.41 (2)°. The organic cations inter­act with the Br^−^ anions by N—H⋯Br hydrogen bonds, leading to the formation of chains parallel to the *b* axis.

## Related literature

For tetra­zole derivatives, see: Zhao *et al.* (2008 ▶); Fu *et al.* (2008 ▶, 2009 ▶). For the crystal structures and properties of related compounds, see: Fu *et al.* (2007 ▶, 2009 ▶); Fu & Xiong (2008 ▶).
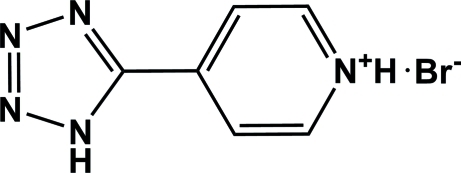


## Experimental

### 

#### Crystal data

C_6_H_6_N_5_^+^·Br^−^
*M* *_r_* = 228.07
Monoclinic, 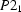

*a* = 4.8688 (10) Å
*b* = 7.6850 (15) Å
*c* = 11.174 (2) Å
β = 92.38 (3)°
*V* = 417.73 (14) Å^3^
*Z* = 2
Mo *K*α radiation
μ = 4.87 mm^−1^
*T* = 298 K
0.30 × 0.05 × 0.05 mm

#### Data collection

Rigaku Mercury2 diffractometer
Absorption correction: multi-scan (*CrystalClear*; Rigaku, 2005 ▶) *T*_min_ = 0.910, *T*_max_ = 1.000
4378 measured reflections
1897 independent reflections
1738 reflections with *I* > 2σ(*I*)
*R*_int_ = 0.033

#### Refinement

*R*[*F* ^2^ > 2σ(*F* ^2^)] = 0.029
*wR*(*F* ^2^) = 0.058
*S* = 1.08
1897 reflections
109 parameters
1 restraint
H-atom parameters constrained
Δρ_max_ = 0.41 e Å^−3^
Δρ_min_ = −0.28 e Å^−3^
Absolute structure: Flack (1983) ▶, 869 Friedel pairs
Flack parameter: 0.045 (11)

### 

Data collection: *CrystalClear* (Rigaku, 2005 ▶); cell refinement: *CrystalClear*; data reduction: *CrystalClear*; program(s) used to solve structure: *SHELXS97* (Sheldrick, 2008 ▶); program(s) used to refine structure: *SHELXL97* (Sheldrick, 2008 ▶); molecular graphics: *SHELXTL* (Sheldrick, 2008 ▶); software used to prepare material for publication: *SHELXTL*.

## Supplementary Material

Crystal structure: contains datablocks I, global. DOI: 10.1107/S1600536810050658/pk2287sup1.cif

Structure factors: contains datablocks I. DOI: 10.1107/S1600536810050658/pk2287Isup2.hkl

Additional supplementary materials:  crystallographic information; 3D view; checkCIF report

## Figures and Tables

**Table 1:** Hydrogen-bond geometry (Å, °)

*D*—H⋯*A*	*D*—H	H⋯*A*	*D*⋯*A*	*D*—H⋯*A*
N1—H1*A*⋯Br1^i^	0.86	2.35	3.210 (3)	178
N2—H2*A*⋯Br1^ii^	0.86	2.37	3.193 (3)	160

Symmetry codes: (i) 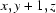; (ii) 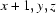.

**Fig. 1.**
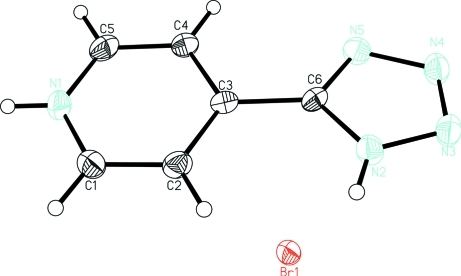
A view of the title compound with the atomic numbering scheme. Displacement ellipsoids are drawn at the 30% probability level.

**Fig. 2.**
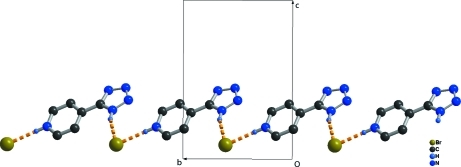
The crystal packing of the title compound, showing the one-dimensional hydrogen-bonded chain. H atoms not involved in hydrogen bonding (dashed line) have been omitted for clarity.

## supplementary crystallographic information

### Comment

Tetrazole compounds have attracted attention as phase transition dielectric materials for application in micro-electronics and memory storage. With the purpose of obtaining phase transition crystals of 4-(1*H*-tetrazol-5-yl)pyridine salts, its interaction with various acids has been studied and a series of new materials have been made with this organic molecule (Zhao *et al.*, 2008; Fu *et al.*, 2008; Fu *et al.*, 2007; Fu & Xiong 2008). In this paper, we describe the crystal structure of the title compound, 4-(1*H*-tetrazol-5-yl)pyridinium bromide.

In the title compound (Fig.1), the pyridine N atoms are protonated. The pyridine and tetrazole rings are nearly coplanar and only twisted from each other by a dihedral angle of 6.41 (2)°. The geometric parameters of the tetrazole rings are comparable to those in related molecules (Zhao *et al.*, 2008; Fu *et al.*, 2009).

In the crystal structure, the organic cations are connected by the Br^-^ anions through two type of N—H···Br hydrogen bonds, with the N···Br distance of 3.210 (3)Å and 3.193 (3) Å, respectively. Those H-bonds link the ionic species into a one-dimensional chain parallel to the *b* axia (Table 1 and Fig.2).

### Experimental

Isonicotinonitrile (30 mmol), NaN_3_ (45 mmol), NH_4_Cl (33 mmol) and DMF (50 ml) were added in a flask under nitrogen atmosphere and the mixture stirred at 110°C for 20 h. The resulting solution was then poured into ice-water (100 ml), and a white solid was obtained after adding HCl (6 *M*) to pH=6. The precipitate was filtered and washed with distilled water. Colourless block-shaped crystals suitable for X-ray analysis were obtained from the crude product by slow evaporation of a water/HBr (50:1 *v*/*v*) solution.

Permittivity measurement show that there is no phase transition within the temperature range (from 100 K to 400 K), and the permittivity is 6.1 at 1 MHz at room temperature.

### Refinement

All H atoms attached to C and N atoms were fixed geometrically and treated as riding with C–H = 0.93 Å (aromatic) and N–H = 0.86 Å with *U*_iso_(H) =1.2Ueq(C or N).

### Crystal data

**Table:**

C_6_H_6_N_5_^+^·Br^−^	*F*(000) = 224
*M**_r_* = 228.07	*D*_x_ = 1.813 Mg m^−^^3^
Monoclinic, *P*2_1_	Mo *K*α radiation, λ = 0.71073 Å
Hall symbol: P 2yb	Cell parameters from 1897 reflections
*a* = 4.8688 (10) Å	θ = 3.2–27.5°
*b* = 7.6850 (15) Å	µ = 4.87 mm^−^^1^
*c* = 11.174 (2) Å	*T* = 298 K
β = 92.38 (3)°	Block, colorless
*V* = 417.73 (14) Å^3^	0.30 × 0.05 × 0.05 mm
*Z* = 2	

### Data collection

**Table:**

Rigaku Mercury2 diffractometer	1897 independent reflections
Radiation source: fine-focus sealed tube	1738 reflections with *I* > 2σ(*I*)
graphite	*R*_int_ = 0.033
Detector resolution: 13.66 pixels mm^-1^	θ_max_ = 27.5°, θ_min_ = 3.2°
ω scan	*h* = −6→6
Absorption correction: multi-scan (*CrystalClear*; Rigaku, 2005)	*k* = −9→9
*T*_min_ = 0.910, *T*_max_ = 1.000	*l* = −14→14
4378 measured reflections	

### Refinement

**Table:**

Refinement on *F*^2^	Secondary atom site location: difference Fourier map
Least-squares matrix: full	Hydrogen site location: inferred from neighbouring sites
*R*[*F*^2^ > 2σ(*F*^2^)] = 0.029	H-atom parameters constrained
*wR*(*F*^2^) = 0.058	*w* = 1/[σ^2^(*F*_o_^2^) + (0.0135*P*)^2^] where *P* = (*F*_o_^2^ + 2*F*_c_^2^)/3
*S* = 1.08	(Δ/σ)_max_ < 0.001
1897 reflections	Δρ_max_ = 0.41 e Å^−^^3^
109 parameters	Δρ_min_ = −0.28 e Å^−^^3^
1 restraint	Absolute structure: Flack (1983), 869 Friedel pairs
Primary atom site location: structure-invariant direct methods	Flack parameter: 0.045 (11)

### Special details

**Table:**

Geometry. All e.s.d.'s (except the e.s.d. in the dihedral angle between two l.s. planes) are estimated using the full covariance matrix. The cell e.s.d.'s are taken into account individually in the estimation of e.s.d.'s in distances, angles and torsion angles; correlations between e.s.d.'s in cell parameters are only used when they are defined by crystal symmetry. An approximate (isotropic) treatment of cell e.s.d.'s is used for estimating e.s.d.'s involving l.s. planes.
Refinement. Refinement of *F*^2^ against ALL reflections. The weighted *R*-factor *wR* and goodness of fit *S* are based on *F*^2^, conventional *R*-factors *R* are based on *F*, with *F* set to zero for negative *F*^2^. The threshold expression of *F*^2^ > σ(*F*^2^) is used only for calculating *R*-factors(gt) *etc*. and is not relevant to the choice of reflections for refinement. *R*-factors based on *F*^2^ are statistically about twice as large as those based on *F*, and *R*- factors based on ALL data will be even larger.

### Fractional atomic coordinates and isotropic or equivalent isotropic displacement parameters (Å^2^)

**Table:**

	*x*	*y*	*z*	*U*_iso_*/*U*_eq_	
Br1	0.54779 (5)	0.11064 (8)	0.91219 (2)	0.04195 (11)	
C1	0.9198 (7)	0.6502 (5)	0.8550 (3)	0.0389 (10)	
H1	1.0123	0.6938	0.9230	0.047*	
C2	0.9881 (7)	0.4908 (5)	0.8113 (3)	0.0357 (8)	
H2	1.1263	0.4251	0.8494	0.043*	
C6	0.9049 (6)	0.2572 (4)	0.6591 (3)	0.0298 (7)	
N1	0.7207 (5)	0.7439 (4)	0.8003 (2)	0.0396 (7)	
H1A	0.6786	0.8433	0.8297	0.048*	
C3	0.8491 (6)	0.4280 (4)	0.7096 (3)	0.0285 (6)	
N3	1.0851 (7)	0.0009 (4)	0.6315 (3)	0.0436 (8)	
N2	1.1026 (5)	0.1460 (4)	0.6956 (3)	0.0367 (8)	
H2A	1.2233	0.1657	0.7524	0.044*	
C5	0.5854 (8)	0.6883 (5)	0.7017 (3)	0.0393 (10)	
H5	0.4496	0.7575	0.6651	0.047*	
N4	0.8778 (6)	0.0253 (4)	0.5546 (3)	0.0424 (8)	
C4	0.6457 (7)	0.5292 (5)	0.6541 (3)	0.0355 (9)	
H4	0.5513	0.4897	0.5853	0.043*	
N5	0.7617 (6)	0.1826 (4)	0.5708 (3)	0.0386 (8)	

### Atomic displacement parameters (Å^2^)

**Table:**

	*U*^11^	*U*^22^	*U*^33^	*U*^12^	*U*^13^	*U*^23^
Br1	0.04736 (18)	0.04135 (19)	0.03641 (18)	0.0106 (2)	−0.00715 (13)	−0.0064 (2)
C1	0.0434 (17)	0.039 (3)	0.0339 (17)	0.0035 (15)	−0.0039 (15)	−0.0041 (15)
C2	0.0334 (18)	0.037 (2)	0.036 (2)	0.0074 (15)	−0.0027 (16)	0.0039 (16)
C6	0.0299 (16)	0.0273 (17)	0.0321 (18)	0.0047 (13)	−0.0010 (14)	0.0065 (14)
N1	0.0515 (17)	0.0286 (15)	0.0393 (16)	0.0081 (13)	0.0075 (15)	−0.0035 (13)
C3	0.0276 (15)	0.0316 (17)	0.0263 (16)	−0.0003 (12)	−0.0003 (13)	0.0061 (13)
N3	0.0523 (19)	0.0330 (18)	0.045 (2)	0.0094 (15)	−0.0047 (17)	−0.0071 (14)
N2	0.0338 (13)	0.038 (3)	0.0371 (14)	0.0048 (13)	−0.0064 (12)	−0.0073 (14)
C5	0.039 (2)	0.037 (2)	0.041 (2)	0.0084 (15)	−0.0007 (18)	0.0101 (17)
N4	0.0475 (19)	0.0360 (18)	0.0431 (19)	0.0054 (16)	−0.0046 (17)	−0.0102 (15)
C4	0.039 (2)	0.0355 (19)	0.032 (2)	0.0060 (15)	−0.0059 (16)	0.0035 (15)
N5	0.0411 (17)	0.0381 (18)	0.0357 (17)	0.0011 (14)	−0.0099 (15)	−0.0036 (13)

### Geometric parameters (Å, °)

**Table:**

C1—N1	1.335 (4)	N1—H1A	0.8600
C1—C2	1.365 (5)	C3—C4	1.386 (5)
C1—H1	0.9300	N3—N4	1.312 (5)
C2—C3	1.385 (5)	N3—N2	1.326 (4)
C2—H2	0.9300	N2—H2A	0.8600
C6—N5	1.315 (4)	C5—C4	1.370 (4)
C6—N2	1.338 (4)	C5—H5	0.9300
C6—C3	1.459 (4)	N4—N5	1.350 (3)
N1—C5	1.330 (5)	C4—H4	0.9300
			
N1—C1—C2	120.2 (3)	C4—C3—C6	118.2 (3)
N1—C1—H1	119.9	N4—N3—N2	105.3 (3)
C2—C1—H1	119.9	N3—N2—C6	110.1 (3)
C1—C2—C3	119.2 (3)	N3—N2—H2A	125.0
C1—C2—H2	120.4	C6—N2—H2A	125.0
C3—C2—H2	120.4	N1—C5—C4	120.1 (4)
N5—C6—N2	107.6 (3)	N1—C5—H5	120.0
N5—C6—C3	125.5 (3)	C4—C5—H5	120.0
N2—C6—C3	126.8 (3)	N3—N4—N5	110.8 (3)
C5—N1—C1	122.1 (3)	C5—C4—C3	119.2 (4)
C5—N1—H1A	119.0	C5—C4—H4	120.4
C1—N1—H1A	119.0	C3—C4—H4	120.4
C2—C3—C4	119.2 (3)	C6—N5—N4	106.2 (3)
C2—C3—C6	122.6 (3)		
			
N1—C1—C2—C3	0.2 (5)	C3—C6—N2—N3	177.7 (3)
C2—C1—N1—C5	−1.0 (6)	C1—N1—C5—C4	1.0 (5)
C1—C2—C3—C4	0.5 (5)	N2—N3—N4—N5	−1.1 (4)
C1—C2—C3—C6	−178.4 (3)	N1—C5—C4—C3	−0.2 (5)
N5—C6—C3—C2	172.4 (3)	C2—C3—C4—C5	−0.6 (5)
N2—C6—C3—C2	−5.2 (5)	C6—C3—C4—C5	178.4 (3)
N5—C6—C3—C4	−6.6 (5)	N2—C6—N5—N4	−0.4 (4)
N2—C6—C3—C4	175.8 (3)	C3—C6—N5—N4	−178.4 (3)
N4—N3—N2—C6	0.8 (4)	N3—N4—N5—C6	1.0 (4)
N5—C6—N2—N3	−0.3 (4)		

### Hydrogen-bond geometry (Å, °)

**Table:**

*D*—H···*A*	*D*—H	H···*A*	*D*···*A*	*D*—H···*A*
N1—H1A···Br1^i^	0.86	2.35	3.210 (3)	178.
N2—H2A···Br1^ii^	0.86	2.37	3.193 (3)	160.

Symmetry codes: (i) *x*, *y*+1, *z*; (ii) *x*+1, *y*, *z*.
